# The biotechnological potential of bacterial extracellular polymeric substances in lead biosorption

**DOI:** 10.3389/fmicb.2025.1650222

**Published:** 2025-11-26

**Authors:** Pfariso Maumela, Mahloro Serepa-Dlamini

**Affiliations:** Department of Biotechnology and Food Technology, Faculty of Science, University of Johannesburg, Johannesburg, South Africa

**Keywords:** *Bacillus*, carboxyl, FTIR, lead, hydrophobicity

## Abstract

**Introduction:**

Extracellular polymeric substances are composed of a diverse range of functional groups, thereby making a strong case for their consideration as biosorbents in heavy metal bioremediation. This study, therefore, assessed strategies to enhance the biotechnological potential of extracellular polymeric substances produced by an endophytic bacterium, *Bacillus* MHSD_36.

**Methods:**

Design of experiments were used to optimize the yield of extracellular polymeric substances from *Bacillus* MHSD_36. A mixture design was subsequently used to develop a cocktail of EPS and hydrophobicity components for the optimal biosorption of lead.

**Results:**

The production of the EPS, from MHSD_36, was optimized through lead induction at a concentration and time of 5.23 mg/L and 10.75 h, respectively. The optimum yield was 1.65 g/L EPS. The use of garden compost, as an alternative growth medium, was sufficient to give an EPS yield (1.15 g/L) comparable to sucrose based medium (1.25 g/L) under optimal induction conditions. The EPS from the *Bacillus* MHSD_36 had a Pb biosorption of 14.24%. However, a mixture of EPS with the hydrophobicity components significantly enhanced the Pb biosorption. The optimal proportion for the mixture was estimated to be 0.25 and 0.75, respectively with a maximum Pb biosorption of 95.8%. The acid recovery of EPS after the biosorption was effective to recover and recycle EPS in heavy metal biosorption.

**Discussion:**

The production of EPS using garden compost and the subsequent recovery after biosorption of heavy metal offers a sustainable approach for the biotechnological application of bacterial EPS in environmental bioremediation.

## Introduction

Heavy metal contamination due to industrialization has a major impact on human health and the biosphere (Upadhyay, 2022). Remediation can be achieved through physical, chemical or biological methods (Dhaliwal et al., 2020; Rajendran et al., 2022). Biological methods such as microbial based biosorption have gained significant attention as an alternative and sustainable method of heavy metal bioremediation (Dave et al., 2020; Priya et al., 2022). Microbial biosorption is easy to operate, has high metal binding efficiency and produces no toxic waste residues (Elgarahy et al., 2021). Bacteria have been shown to have a sorption capacity of approximately 200 mg/g for heavy metals such Pb (Lead) and Hg (Mercury) (Mohapatra et al., 2019). Biosorption can be achieved with dead or live bacterial cells and is facilitated by physicochemical interactions such as ion exchange, complexation, precipitation, adsorption, and absorption (Dave et al., 2020; Elgarahy et al., 2021). The ability of bacteria to bind to metal ions is attributed to the synthesis of extracellular polymeric substances (EPS) (Sonkar et al., 2024).

EPS are negatively charged polymers because they are composed of ionizable functional groups such as carboxyl, sulfate, phosphate, hydroxyl, amine, and sulfhydryl groups (Li et al., 2022). Furthermore, EPS are diverse polymers with a range of potential industrial applications from pharmaceutical, food, textile, agriculture, to packaging (Tiwari et al., 2020; Behera et al., 2021). Thus, the synthesis and production of EPS, which entails the development of production and recovery methods, is of biotechnological significance. Bacterial EPS production is performed in liquid cultures supplemented with a carbon and nitrogen source (Liu et al., 2022; Rath et al., 2022). The EPS are subsequently recovered from the broth through precipitation, following the removal of microbial cells and proteins (Siddharth et al., 2021). Dialysis and lyophilisation can be applied for the purification of the recovered EPS (Feng et al., 2019).

Carbon is an essential nutrient and provides energy for cell growth (Liu et al., 2022; Rath et al., 2022). The widely used carbon sources for EPS production are starch, sucrose, glucose, maltose, galactose, lactose and fructose (Song et al., 2020). Glucose and sucrose have, however, been shown to result in high EPS yields in bacterial endophytes (Liu et al., 2017). Li et al. (2012) achieved an EPS yield of 22% and a concentration of 14 g/L with the fungal *Berkleasmium* sp. Dzf12. Furthermore, nitrogen sources play an important role in EPS production because they support cell growth as a source of nitrogen for nucleic acid and protein synthesis (Liu et al., 2022). Cultivation conditions such as pH, temperature, aeration and time have been reported to affect EPS production (Jyoti et al., 2024).

Bacterial endophytes (Fuentes-Quiroz et al., 2024) synthesize extracellular polymeric substances (EPS) which are involved in cell aggregation, biofilm formation and nutrient uptake (Ajijah et al., 2023). Moreover, bacterial endophytes of *Bacillus* sp dominate the plant microbiota (Susanti et al., 2024) and can use complex carbon and nitrogen sources, from their plant host and soil, for their growth (Saikia and Thakur, 2024). *Bacillus* sp survives in heavy metal contaminated environment through metal biosorption on the cells walls or externally by producing EPS (Sraboni et al., 2024). Therefore, the overall objective of this study was to evaluate the heavy metal biosorption capacity of EPS produced by an endophytic bacteria strain *Bacillus* MHSD_36. The study investigated the impact of heavy metal stress, using lead (Pb) as a model because it is a prominent carcinogen found in the environment (Aendo et al., 2022), on the EPS yield of *Bacillus* MHSD_36. The potential of using garden compost as a nutrient source for EPS production was also explored. The study investigated the impact of simultaneously using EPS and cell surface hydrophobicity components (hydrophobicity components) on heavy metal biosorption efficiency. Finally, the study investigated the potential of recovering and re-using the EPS in multiple rounds of heavy metal biosorption.

## Materials and methods

### Bacterial strain maintenance

A 30% glycerol stock of the bacterial culture of *Bacillus* MHSD_36 (NCBI GenBank accession JAVBIS000000000) was plated on nutrient agar and incubated for 24 h at 28 °C, for routine culture maintenance.

### Extracellular polymeric substances production and recovery from culture broth

The bacterial strain was first streaked on nutrient agar (NA) plates supplemented with sucrose 5% (w/v) and incubated at 37 °C for 72 h. Nutrient broth was inoculated with a single colony of the bacterial culture and incubated at 30 °C with agitation at 150 rpm for 24 h to prepare the pre-inoculum. One mL of the pre-inoculum was added into 500 mL fresh broth with 10% sucrose and incubated for 72 h at 30 °C agitating at 150 rpm. For EPS production in compost, the nutrient broth with 10% sucrose was replaced with 25% compost. The culture broth was subsequently centrifuged at 8000 rpm and for 15 mins. The supernatant was separated from the cells, mixed with twice the volume of 95% ethanol and incubated at 4 °C overnight for EPS precipitation. The EPS was recovered through centrifugation at 8000 rpm and 4 °C for 30 mins. The recovered EPS was then air dried. The concentration of EPS was determined using the phenol sulfuric assay with glucose as a standard, according to the method of DuBois et al. (1956). Briefly, 600 μL of 95% H_2_SO_4_, 120 μL of 5% phenol and 200 μL of the sample were mixed, heat in a boiling water bath for 5 min, samples cooled, and absorbance determined at 490 nm.

### Experimental design: the impact of heavy metal stress and induction time on EPS production

EPS production was enhanced through exposing the bacterial culture, described in the preceding section, to stress. A five-coded levels central composite design (CCD) was used to determine the impact of Pb concentration and induction time on EPS production. The experiment had 13 runs including 5 center points (Table 1). The factors were in the following range, Pb concentration (0.75, 2, 5, 8, 9.24 mg/L) (Huang et al., 2025) and induction time (1.5, 4, 10, 16, 18.5). The general formula for the response is shown in the equation below:


$$\begin{array}{l} y_{i}=\;\beta_{0}+\;\overset{n}{\underset{i\;=1}{\sum }}\beta_{i}x_{i}\;+\overset{n}{\underset{\;i=1}{\sum }}\beta_{ii}x_{i}^{2}\;+\;\underset{i<j}{\sum }\beta_{ij}x_{i}x_{j}\;+\epsilon \end{array}$$ (1)

where y_i_ is the i^th^ response variable, x_i_ is the i^th^ input parameter, n is the number of input parameters and β_0_, β_i_, β_ii_, β_ij_ are the fixed response, linear, quadratic, and cross products coefficients, respectively. Minitab (Design–Expert, Stat-Ease Inc. Minneapolis, MN, USA) was used for the experimental design and statistical analysis at 5% level of significance.

**Table 1:** Experimental design for the optimization of extracellular polymeric substance yields through lead stress induction.

**Std**	**Run**	**A:Induction time (h)**	**B:Pb concentration (mg/L)**	**EPS volumetric yield (g/L)**
10.00	1.00	10.00	5.00	1.48
9.00	2.00	10.00	5.00	1.67
11.00	3.00	10.00	5.00	1.87
8.00	4.00	10.00	9.24	0.76
7.00	5.00	10.00	0.75	0.50
1.00	6.00	4.00	2.00	1.13
3.00	7.00	4.00	8.00	1.02
5.00	8.00	1.51	5.00	0.63
4.00	9.00	16.00	8.00	1.16
13.00	10.00	10.00	5.00	1.91
12.00	11.00	10.00	5.00	1.31
6.00	12.00	18.49	5.00	1.06
2.00	13.00	16.00	2.00	0.98

### Extraction of cell surface hydrophobicity components

*Bacillus* MHSD_36 was grown in nutrient broth (NB) at 28 °C, 150 rpm for 24 h and hydrophobicity components extracted according to the method of Khalil et al. (2018). Bacterial cells were harvested from the broth by centrifugation at 8000 rpm for 15 min and washed twice with 5 mL of phosphate buffered saline (PBS) pH 7. The washed cells were resuspended in 2 mL of PBS, mixed with an equal volume of chloroform and vortexed for 2 min. The aqueous and organic phases were allowed to separate for 30 min at room temperature (±25 °C). The aqueous phase was separated (hydrophobicity components) from the organic phase and stored until use.

### Heavy metal biosorption

The biosorption studies were performed by combining 1 mL of the biosorbent and 4 mL of de-ionized water containing Pb to give a final volume and Pb concentration of 5 mL and 10 mg/L, respectively. The mixture was incubated at 25 °C with agitating at 150 rpm for 24 h and the concentration of residual Pb determined with Inductively coupled plasma optical emission spectroscopy (ICP-OES). The biosorbent was 5 g/L of EPS.

### Mixture design: interaction between EPS and hydrophobicity components

A {2, 4} simplex lattice design augmented with interior points and centroid (Montgomery, 2017) was used to optimize the proportion of EPS and hydrophobicity components for heavy metal biosorption. The experimental design had a total of 9 runs including 4 center points. The heavy metal biosorption experiments were performed as described in the previous section. The biosorbent was however made up of the different proportions of EPS and hydrophobicity components defined by the lattice design.

### The recovery and re-use of EPS

The potential of salt and acid as EPS recovery agents following heavy metal biosorption was evaluated. The air-dried EPS extract was resuspended in 15 ml deionised water, with 10 mg/L Pb, to give an EPS concentration of 5 g/L. The mixture was incubated at 25 °C with agitating at 150 rpm for 24 h. One ml of the mixture was sampled for the determination of the residual Pb concentration with ICP-OES. For the acid recovery, the pH of the biosorption mixture was adjusted to 3 with nitric acid. The biosorption mixture was treated with aluminum oxide (Al_2_O_3_) to a concentration of 3 mg/L for the salt recovery procedure. The recovery experiments were left standing at room temperature overnight. The EPS was subsequently recovered through centrifugation at 8000 rpm and 4 °C for 30 mins. The recovered EPS was resuspended in 15 ml deionised water and incubated at 25 °C with agitating at 150 rpm for 24 h. The concentration of the residual Pb was determined with ICP-OES.

### Fourier transform infrared spectroscopy analysis of EPS

The chemical composition of the EPS was determined using Fourier transform infrared spectroscopy (FTIR) to gain insight into functional groups important for metal adsorption. The FTIR spectra of the samples were obtained using an FTIR spectrophotometer (Thermo Scientific Smart iTR, Attenuated Total Reflectance, USA).

### Statistical analysis for metal biosorption experiments

All the metal biosorption experiments were performed in triplicates and results were presented in the form of the mean ± SD. The standard deviations were represented in charts as error bars. The significant difference was determined by the analysis of variance (ANOVA) in Microsoft Excel 365. The ANOVA was performed at 5% level of significance.

## Results

### Enhancing the production of EPS through growth under heavy metal stress

**Figure 1:**
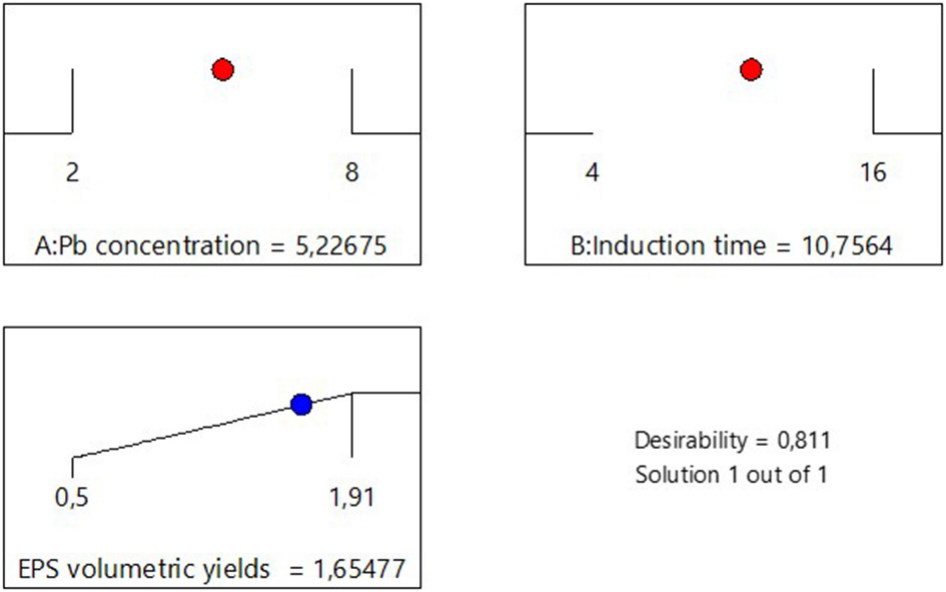
Desirability analysis for the optimization of Pb concentration and induction time in EPS production.

**Figure 2:**
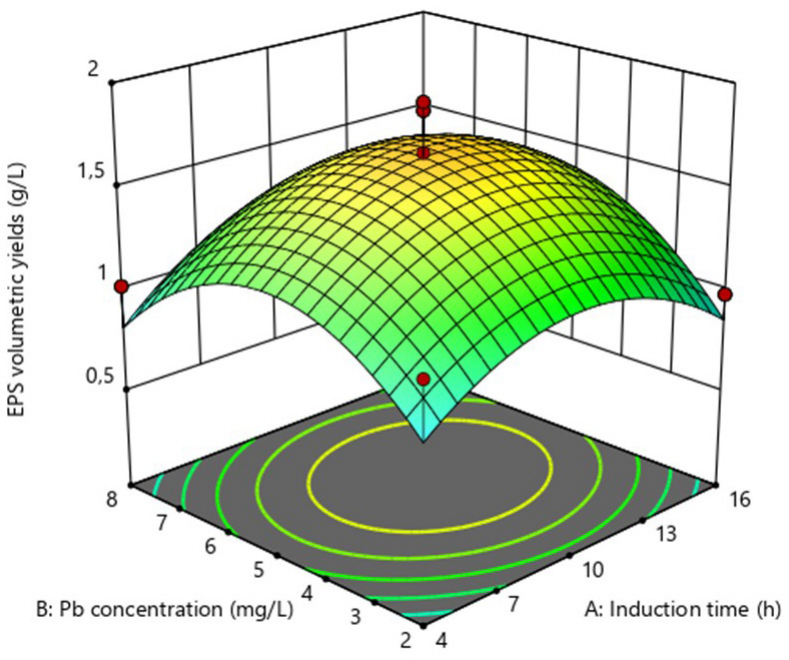
Surface response plot for the optimization of EPS volumetric yields from *Bacillus* MHSD_36.

The impact of Pb induction concentration and time on EPS production by the bacterial strain *Bacillus* MHSD_36 was evaluated using the response surface methodology. Table 1 is a summary of the statistical design and corresponding experimental values of the EPS volumetric yields, ranging between 0.5-1.91 mg/L. The optimal Pb induction concentration and time were 5.23 mg/L and 10.75 h, respectively, with an optimum volumetric yield of 1.65 g/L EPS (Figure 1). ANOVA (Table 2) shows that the R^2^ and adjusted R^2^ value is 0.7712 and 0.6972, respectively. Thus, 69.72% of the variation in the EPS yield was attributed to the Pb induction concentration and time. The residual analysis was used to determine the model adequacy and data summarized in residual plots (Supplementary Figure 1). The plots showed that there was no correlation in the residuals of the different runs and the residuals did not corelate to the predicted response. In addition, the normality plot (Supplementary Figure 2) showed that the residuals were normally distributed. The quadratic terms of both concentration (B^2^) and induction time (A^2^) had a *p*-value of < 0.05 (Table 2), illustrating that an initial increase in the Pb induction concentration and time results in an increase in the EPS volumetric yield until a maximum level (Figure 2). However, subsequent increases resulted in a decrease in the EPS yields (Figure 2). Equations 1 and 2 is the coded model equation and actual model equation, respectively, illustrating the mathematical relationship between the EPS yield and Pb induction concentration and time;


$$\begin{array}{l} y=0.0748A+0.0547B+0.0725AB-\;{0.3177A}^{2}-{0.4253B}^{2}\\ \;+\;1.65 \end{array}$$ (2)

$$\begin{array}{l} y=0.1688A+0.4504B+0.004AB-\;{0.0088A}^{2}-{0.0472B}^{2}\\ \;-\;0.4302 \end{array}$$ (3)

where, *y* is the volumetric EPS yield, *A* is the Pb induction time and *B* is the Pb concentration. The regression coefficient data for the coded model equation is summarized in Supplementary Table 1. The negative sign on the quadratic terms further illustrates that the EPS volumetric yield reaches a maximum level before it starts to decline, as the Pb induction concentration and time increase.

**Table 2:** Analysis of variance showing the significant parameters to maximize the yield of EPS.

**Source**	**Sum of squares**	**df**	**Mean square**	***F*-value**	***p*-value**	
Model	1,83	5	0,3669	4,72	0,0332	significant
A-Induction time	0,0447	1	0,0447	5,46	0,4729	
B-Pb concentration	0,0239	1	0,0239	9,72	0,5962	
AB	0,0210	1	0,0210	0,2705	0,6191	
A^2^	0,7024	1	0,7024	9,04	0,0198	
B^2^	1,26	1	1,26	16,18	0,0050	
Residual	0,5442	7	0,0777			
Lack of Fit	0,2833	3	0,0944	1,45	0,3543	not significant
Pure Error	0,2609	4	0,0652			
Cor Total	2,38	12				

### The potential of compost as a nutrient source in EPS production

**Figure 3:**
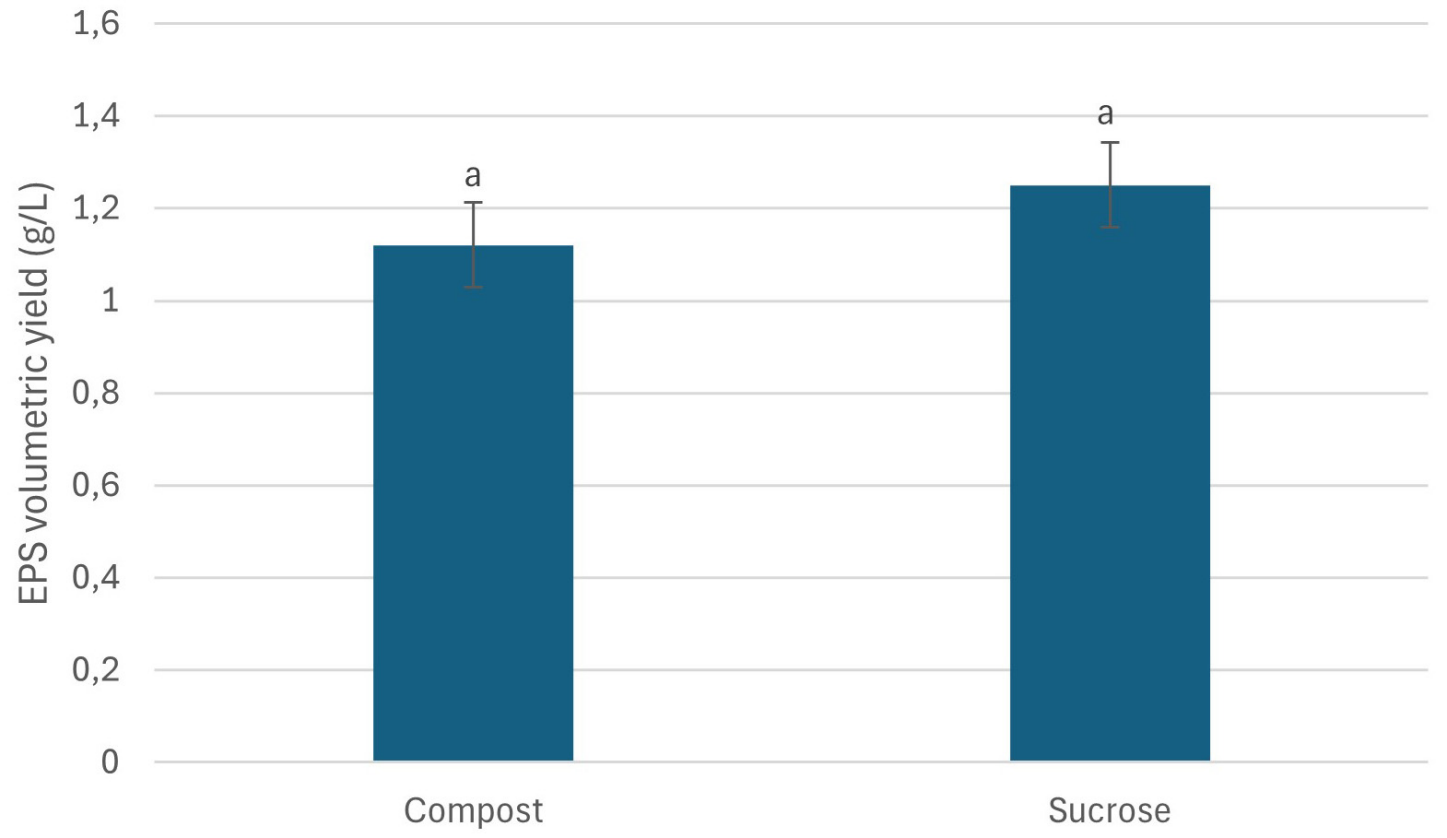
EPS volumetric yields from *Bacillus* MHSD_36 cultured in medium containing compost or/sucrose as carbon sources. The experiments were performed in triplicates and data reported as mean ± standard. The similar letters above the bars show that the difference in the data is not statistically significant.

**Figure 4:**
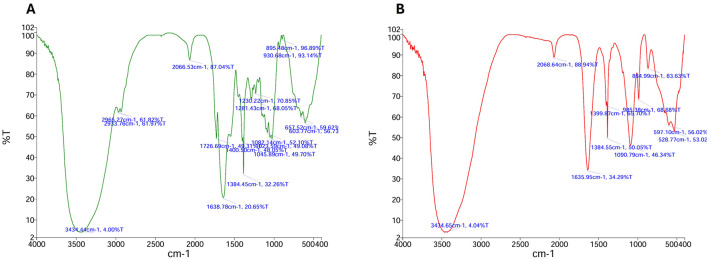
FTIR spectrum of the EPS extracted from the sucrose **(A)** based media and compost media **(B)** cultures of Bacillus MHSD_36.

Garden waste derived compost was evaluated for its potential as a nutritent (carbon and nitrogen) source for EPS production from *Bacillus* sp. MHSD_36. The compost had a fiber and protein content of 72.95 and 8.45, respectively (Table 3). The volumetric yield of EPS was 1.12 g/L when *Bacillus* sp. MHSD_36 was cultured in compost compared to 1.25 g/L in the sucrose and nutrient broth (Figure 3). The functional group composition of the EPS from sucrose and compost cultures is shown in FTIR spectra (Figure 4) showing functional groups in the region of 4,000-400 cm^−1^, characteristics functional groups found in biopolymers. Furthermore, both samples showed a characteristic hydroxyl group corresponding with peak 3434 cm^−1^. Peaks signals 1090 and 930 cm^−1^, characteristic to amine or carboxyl functional groups, were observed for both the EPS and hydrophobicity components (Figure 4). The hydrophobicity components showed a signal at 1045 cm^−1^ which corresponds to a phosphoryl group from phospholipids. The FTIR specta (Figure 4) show that the EPS functional groups such as hydroxyl and phospohryl group were in high abundance while the amine and carboxyl functional groups were in low abundance.

**Table 3:** Proximate composition analysis of the garden compost used in EPS production. The data presented is mean values with ± standard deviation of three replicates.

**Component**	**% composition**
Ash	36.12 + 1.2
Moisture	1.93 + 0.16
Protein	8.45 + 0.50
Fiber	72.95 + 1.96

### The recovery and re-use of EPS for metal biosorption

**Figure 5:**
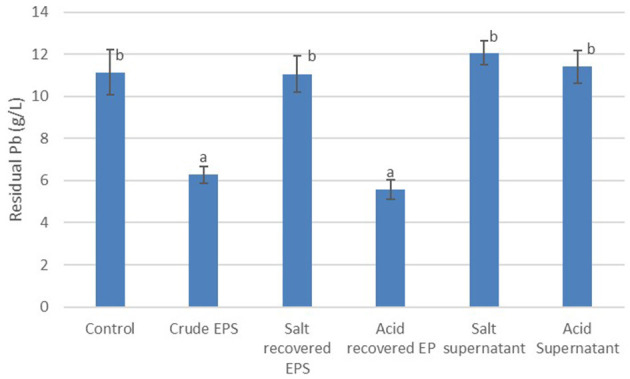
Pb recovery using crude EPS, acid recovered/recycled EPS and salt recovered/recycled EPS. The experiments were performed in triplicates and data reported as mean ± standard deviation. Different letters above the bars indicate statistically significant difference.

The potential for the recovery and re-use of EPS in metal biosorption was evaluated by assaying the ability of acid and salt to recover the EPS following Pb biosorption. The recovered EPS was subsequently used in a second round of Pb biosorption. Figure 5 is a summary of the residual Pb following treatment with crude EPS from *Bacillus* MHSD_36, as well as acid and salt recovered EPS. The data illustrates that acid was suitable for the precipitation and recovery of EPS and subsequent re-use in heavy metal biosorption (Figure 5). There was no significant difference in the residual Pb between the crude EPS (6.27 mg/L) and acid recovered EPS (5.57 mg/L). The supernatant from the acid and salt precipitation assays was mixed with ethanol, following the recovery of the EPS, to further confirm the ability of acid and salt to precipitate the EPS. Figure 5 shows that the residual Pb concentration following the incubation of the ethanol extract with a Pb solution (11.41 and 12.07 mg/L, respectively) was not significantly different from control without a biosorbent (11.13 mg/L).

### Evaluating the synergy between EPS and hydrophobicity components in metal biosorption

**Figure 6:**
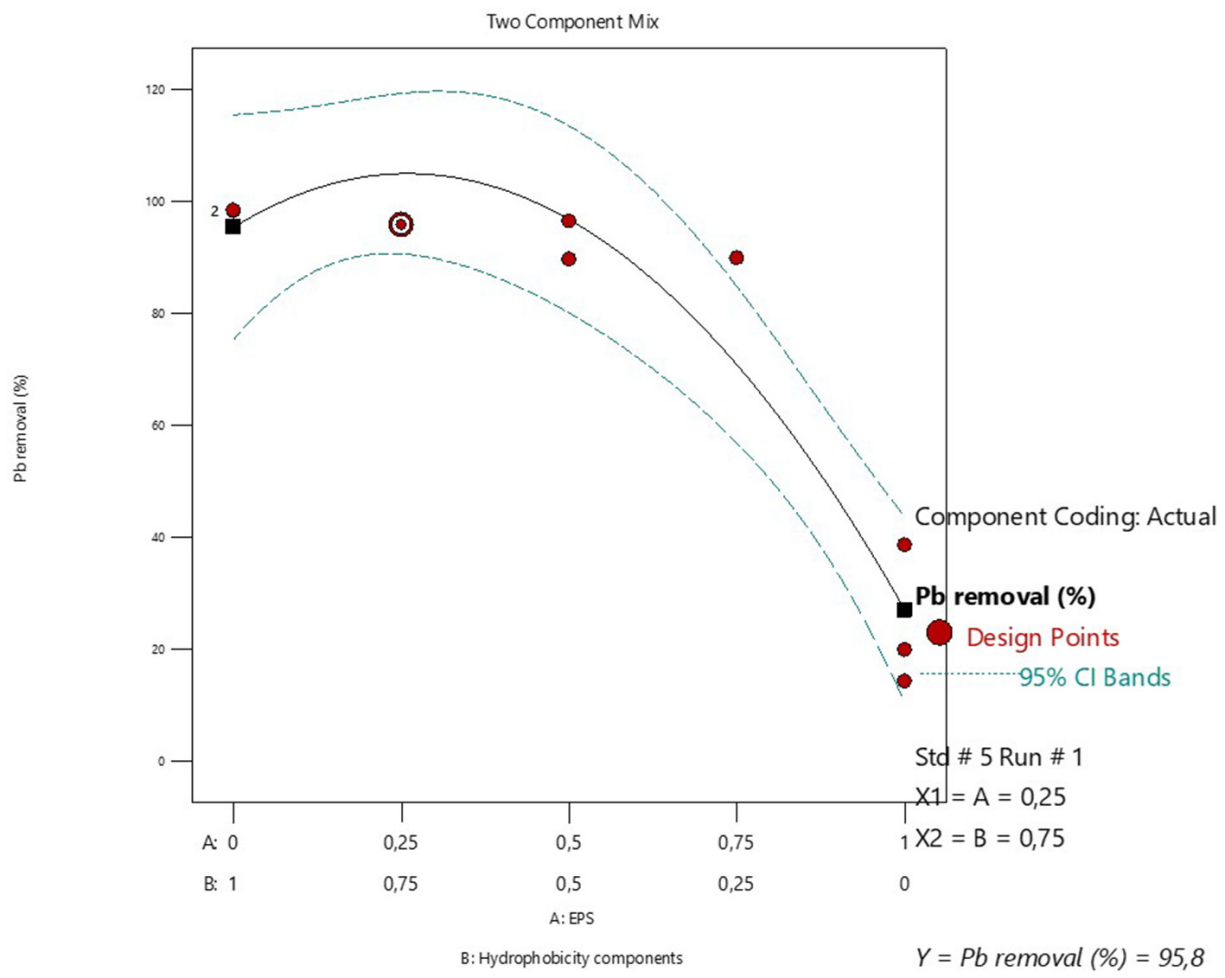
Mixture design for optimizing the proportions of EPS and hydrophobicity components for maximum Pb adsorption and recovery.

Table 4 is a summary of the augmented simplex lattice mixture design used to determine the optimum proportions of EPS and hydrophobicity components for the biosorption of Pb metal ions. The data shows that simultaneouly applying the EPS and hydrophobicity components significantly enhanced the Pb biosorption capacity of the former (Table 4). The lowest Pb biosorption (14.24%) was observed when when the EPS was used without the hydrophobicity components. The partial replacement of EPS by a propotion of 0.25 significantly improved the Pb biosorption to 89.88%. The use of equal proportions of EPS (0.5) and hydrophobicity components (0.5) resulted in the highest observed Pb biosorption of 96.48%. The optimal proportion of EPS and hydrophobicity components was estimated to be 0.25 and 0.75, respectively, with a maximum Pb biosorption of 95.8% (Figure 6).

**Table 4:** Mixture design to determine the optimal proportion of EPS and hydrophobicity components for Pb biosorption.

**Run**	**EPS**	**Hydrophobicity components**	**Pb removal %**
1	0,5	0,5	89,64
2	0,25	0,75	95,8
3	1	0	19,88
4	1	0	14,24
5	0,5	0,5	96,48
6	1	0	38,6
7	0,75	0,25	89,88
8	0	1	98,4
9	0	1	98,28

The ANOVA for the mixture design (Table 5) shows that both the linear and interactive terms of the mixture model were statistically significant. The statistically significant interactive term (AB) is a further indication of the synergistic interaction between EPS and hydrophobicity components in Pb biosorption. In addition, the *R*^2^ and adjusted *R*^2^ value is 0.9164 and 0.8885, respectively, thereby inferring that 88.85% variation in Pb biosorption is attributable to EPS and hydrophobicity components. The residual analyis (Supplementary Figure 3) shows that the residuals were independent and that no correlation existed between the residuals and predicted response. The residuals also had normal distribution (Supplementary Figure 4). The coded model Equation 4, based on the regresssion analysis (Supplementary Figure 3) is shown below;


$$\begin{array}{c} y=27.01A+95.42B+142.24AB \end{array}$$ (4)

where *y* is the Pb removal %, *A* is the EPS proportion and *B* is the hydrophobicity components proportion.

**Table 5:** Analysis of variance for the significant factors in the model for the biosorption of Pb with a cocktail of EPS and hydrophobicity components.

**Source**	**Sum of squares**	**df**	**Mean square**	***F*-value**	***p*-value**	
Model	9481,89	2	4740,94	32,87	0,0006	significant
^(1)^Linear Mixture	7287,06	1	7287,06	50,52	0,0004	
AB	2194,83	1	2194,83	15,22	0,0080	
Residual	865,42	6	144,24			
Lack of Fit	516,80	2	258,40	2,96	0,1623	not significant
Pure Error	348,62	4	87,15			
Cor Total	10347,31	8				

## Discussion

Microbial cells produce EPS as a mechanism to cope with stress including antimicrobial compounds and toxic heavy metals (Li et al., 2022). EPS are thus, a biopolymer of biotechnology and bioprocess significance. Although, EPS have potential biotechnological application in heavy metal bioremediation, there is a paucity in bioprocessing development for the production and application of EPS at industrial scale. This study demostrated that the yields of EPS can be enhanced in liquid cultures through the growth of *Bacillus* MHSD_36 under heavy metal stress (Table 1). The highest volumetric yield (1.65 g/L EPS) was attained through Pb (5.23 mg/L) induction at 10.75 h (Figure 1). Zheng et al. (2008) reported a maximum volumetric yield of 0.8 g/L, equivalent to a substrate productivity of 0.04 g/g of sucrose for *Bacillus sp* MBFF19. A study of Xiong et al. (2010) reported a volumetric yield of 2.93 g/L from *Bacillus licheniformis* CGMCC 2876. The EPS were dominated by hydroxyl, carboxylate and ester functional groups (Xiong et al., 2010). This comparison illustrates the influence of extrinsic factors on EPS production (Jyoti et al., 2024). Morover, EPS production from *Bacillus* MHSD_36 was dependant on the growth phase. This is supported by a study of Ibarburu et al. (2007) which reported that the production of EPS from *Oenococcus oeni* began in the exponential growth phase. Furthermore, stress has been reported to induce and enhance the production of EPS in bacteria (Sengupta et al., 2018). The experimental data illustrated that the induction of MHSD_36 with Pb at mid log-phase (10h) (Figure 2) resulted in maximum EPS yield. The results from this study are congruent with the study of Mohite et al. (2018), which illustrated that the presence of heavy metal enhanced the production of EPS by *Pantoea agglomerans*. The addition of heavy metals, Cd and Cr, has also been shown to significantly increase the EPS content in the range of 36.2 to 60.67 mg/g dry cell (Zeng et al., 2020). Furthermore, increasing the metal concentration above 8 and 30 mg/L for Cd and Cr, respectively, resulted in a significant drop in the EPS content (Zeng et al., 2020). A similar trend was observed in the current study (Figure 2). Thus, it is apparent that excessively high heavy metal concentration are toxic to the bacteria and have a negative impact on the EPS yields (Mohite et al., 2018; Zeng et al., 2020). The observed experimental data also illustrate that EPS production in *Bacillus* MHSD_36 is growth related. Thus, increasing the biomass yields can significantly increase the EPS volumetric yield ultimately making the process to be economical. This strategy of applying heavy metal stress to induce the production of EPS and enhance their yield in liquid culture is further supported by the study of Zeng et al. (2020). The study reported that the production and secretion of EPS from *Bacillus* sp. S3 was stimulated by the presence of cadmiun, copper and chromium metal ions (Zeng et al., 2020).

The use of synthetic growth media is an expensive production technology for the EPS production. Garden waste, therefore, offers an abundant and renewable alternative source of carbon as well as nitrogen (Wenting et al., 2021; Liu et al., 2023). Currently there are limited technologies for the recycling or valorisation of garden which is consequently a burden to municipal landfills (Liu et al., 2023). Thus, a valorisation strategy has the potential to reduce municipal landfill managemrnt cost by 37-50% and greenhouse gas emission by 40% (Jalalipour et al., 2025). Garden waste derived compost was shown to be a potential nutrient (carbon and nitrogen) source for EPS production from *Bacillus* sp. MHSD_36. This is attributable to the high fiber and protein content of the compost, 72.95 and 8.45 %, respectively, (Table 3). The experimental data illustrared that the volumetric yield of EPS (1.12 g/L) from *Bacillus* sp. MHSD_36 cultured in compost was comparable to the sucrose based media (Figure 3). Moreover, the use of compost as the culture media did not alter the fucntional properties of the EPS (Figure 4). The functional group composition of the EPS ranged in the 4000-400 cm^−1^ region of the FTIR spectra (Figure 4). The observed spectrum in Figure 4 are characteristics functional groups from EPS polymers (Li et al., 2022). In addition, the EPS extracts from the compost and sucrose cultures samples were composed of hydroxyl groups which corresponds to peak 3434 cm^−1^ (Figure 4). The presence of hydroxyl groups is charateristics of EPS which are mainly composed of carbohydrates including glucose, fructose, galactose, mannose and arabinose. The monomers are rich in ionizable functional groups; hydroxyl and carboxyl groups (Li et al., 2022) which have been shown to be responsible for metal bisoption (Cui et al., 2020). The signal at 1090 and 930 cm^−1^ for EPS (Figure 4) could be attributed to the presence of amine or carboxyl functional groups. The signal at 1045 cm^−1^ could be attributed phosphoryl group from phospholipids. Mohite et al. (2018) reported that the changes in the infrared spectrum absorption bands, of bacterial cells treated with Pb and Cr, was attributed to the ionization of functional groups. The identified functional groups entailed hydroxyl, amino, carboxyl, and sulphonate, which consequently bind to the heavy metals.

The biotechnological potential of using microbial EPS can be improved further, through the recycling and the reuse of the EPS as a heavy metal biosorbent. The EPS used in the biosorption of Pb was successfully recovered and subsequently re-used in a second round of Pb biosorption. The use of acid was sufficient to recover the EPS, through precipitation and the recovered EPS was successfully re-used in Pb heavy metal biosorption (Figure 5). The residual Pb was 6.27 mg/L and 5.57 mg/L for the crude and acid recovered EPS, respectively (Figure 5), illustrating that the recovered EPS was effective in Pb biosorption. Acidic conditions have been reported to reduce the biosorption capacity of EPS to heavy metals (Concórdio-Reis et al., 2020; Ahamed et al., 2024). Acidic conditions result in a significant amount of H^+^ in solution, which consequently compete with the heavy metals for adsorption (Wei et al., 2015). Furthermore, the functional groups on the surface of EPS become positively charged under acidic conditions, thereby reducing their heavy metal biosorption capacity (Ahamed et al., 2024). However, under highly acidic conditions, EPS degrade and become more soluble. Thus, the use of acid to recover EPS requires optimisation to avoid degradation and loss of EPS as well as preserve of the efficacy of the EPS in subsequent rounds of metal biosorption (Wu et al., 2020).

Bacterial cell wall hydrophobicity has been illustrated to play an important role in toxic heavy metal resistance (Mat et al., 2021; Maity et al., 2023). Therefore, the simultanaeous application of EPS with hydrophobicity components is an attractive strategy to enhance the Pb biosorption capacity of the EPS. The experimental data showed that a cocktail mixture of the EPS and the bacterial hydrophobicity components significantly enhanced the Pb biosorption capacity (Table 4). The presence of teichoic acids and lipoteichoic acids, which are major cell wall components which contribute to the cell hydrophobicity, (Poxton, 2015) explains the strong ability of the hydrophobicity components in heavy metal biosorpion. Moriwaki et al. (2013) reported that lipoteichoic acid-defective strains of *Bacillus subtilis* had a lower biosorption for rare earth ions compared to wild type strains. The addition of hydrophobicity components in the metal remediation is advantageous due to the presence of lipoteichoic acids which adsorbs metal ions and enhance the aggregation of the biosorbent thereby ensuring rapid recovery (Moriwaki et al., 2013). Moreover, the abundance of phosphate functional groups in the hydrophobicity components has been reported to provide primary binding sites for metal ions which consequently enhances metal biosorption during bioremediation (Huang et al., 2025). Thus the EPS and cell hydrophobicity components mixture, greatly enhanced the biosorption efficacy of Pb due to the carboxyl and phosphate functional groups present in the teichoic and lipoteichoic acids from the hydrophobicity components (Jin et al., 2014). Futhermore the compactness and porosity of the EPS and cell hydrophobicity components can be determined using scanning electron microscopy and compared to that of the EPS to illustrate the robustness of the former in metal biosorption (Shen et al., 2022).

The biotechnological potential of EPS from *Bacillus* MHSD_36 can be enhanced through strategies for the sustainable bioprocess development and optimization of the biosorption efficiency. The culturing of strain MHSD_36 under heavy metal stress was shown to be a potential strategy for enhancing the yield of EPS. The strain can also be cultured using compost, as a nutrient source. The EPS yield obtained in compost are significant and comparable to the yield obtained with sucrose-based media. Moreover, the biosorption efficiency of the EPS was significantly enhanced by combining with hydrophobicity components. The EPS was also shown to be recoverable and reusable following application in biosorption of Pb.

## Data Availability

The data from the Whole Genome Shotgun project has been deposited at NCBI GenBank under the accession JAVBIS000000000, BioSample accession number SAMN36845528, and BioProject accession number PRJNA1002565. The version described in this article s JAVBIS000000000.
