# Supplementary material for: The biotechnological potential of bacterial extracellular polymeric substances in lead biosorption
Source: Front Microbiol. 2025 Nov 26;16:1650222. doi: 10.3389/fmicb.2025.1650222 (PMC12690212; doi:10.3389/fmicb.2025.1650222)
Supplement: Supplementary file 1 [file Supplementary_file_1.docx]

Supplementary data

Table S1: Regression analysis for the coded model of EPS production

| **Factor** | **Coefficient Estimate** | **df** | **Standard Error** | **95% CI Low** | **95% CI High** | **VIF** |
| --- | --- | --- | --- | --- | --- | --- |
| Intercept | 1,65 | 1 | 0,1247 | 1,35 | 1,94 |  |
| A-Induction time | 0,0748 | 1 | 0,0986 | -0,1583 | 0,3079 | 1,0000 |
| B-Pb concentration | 0,0547 | 1 | 0,0986 | -0,1784 | 0,2878 | 1,0000 |
| AB | 0,0725 | 1 | 0,1394 | -0,2571 | 0,4021 | 1,0000 |
| A² | -0,3177 | 1 | 0,1057 | -0,5677 | -0,0678 | 1,02 |
| B² | -0,4253 | 1 | 0,1057 | -0,6752 | -0,1753 | 1,02 |

Table S2: Regression analysis for the coded model of the Pb removal model

| **Component** | **Coefficient Estimate** | **df** | **Standard Error** | **95% CI Low** | **95% CI High** | **VIF** |
| --- | --- | --- | --- | --- | --- | --- |
| A-EPS | 27,01 | 1 | 6,78 | 10,43 | 43,59 | 1,31 |
| B-Hydrophobicity components | 95,42 | 1 | 8,21 | 75,34 | 115,50 | 1,46 |
| AB | 142,24 | 1 | 36,46 | 53,02 | 231,46 | 1,80 |


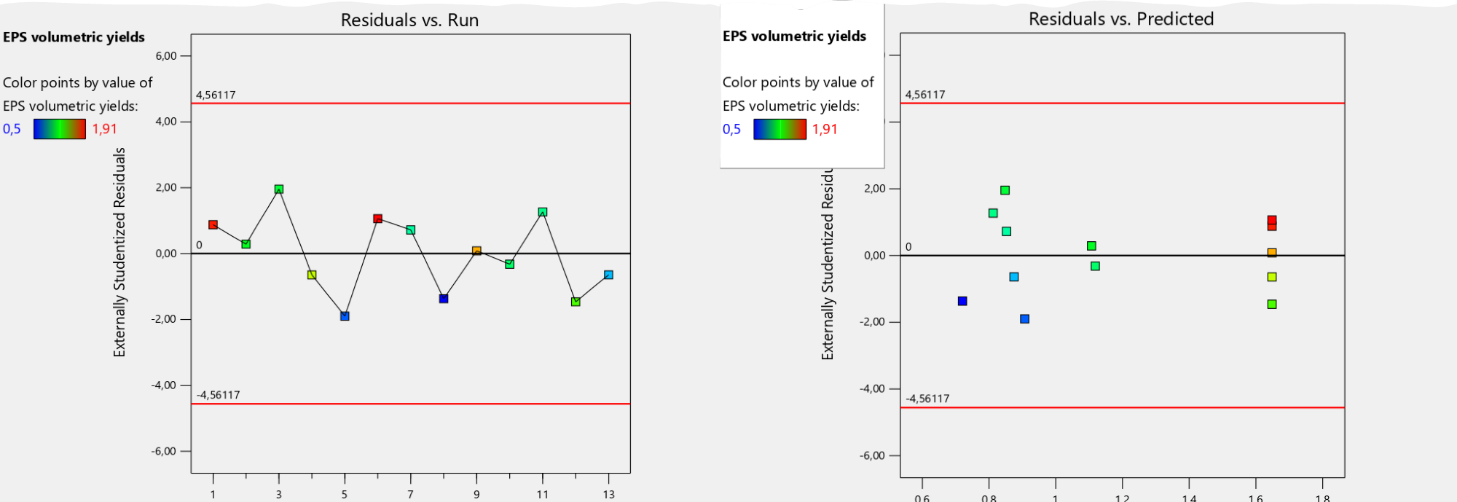


Figure S1: Model adequacy for the EPS production optimisation model. The Residuals vs. Run plots shows that the model data does not violate the independence assumptions on errors. The Residuals vs. Predicted plot shows that the residuals are not related to the predicted responses.


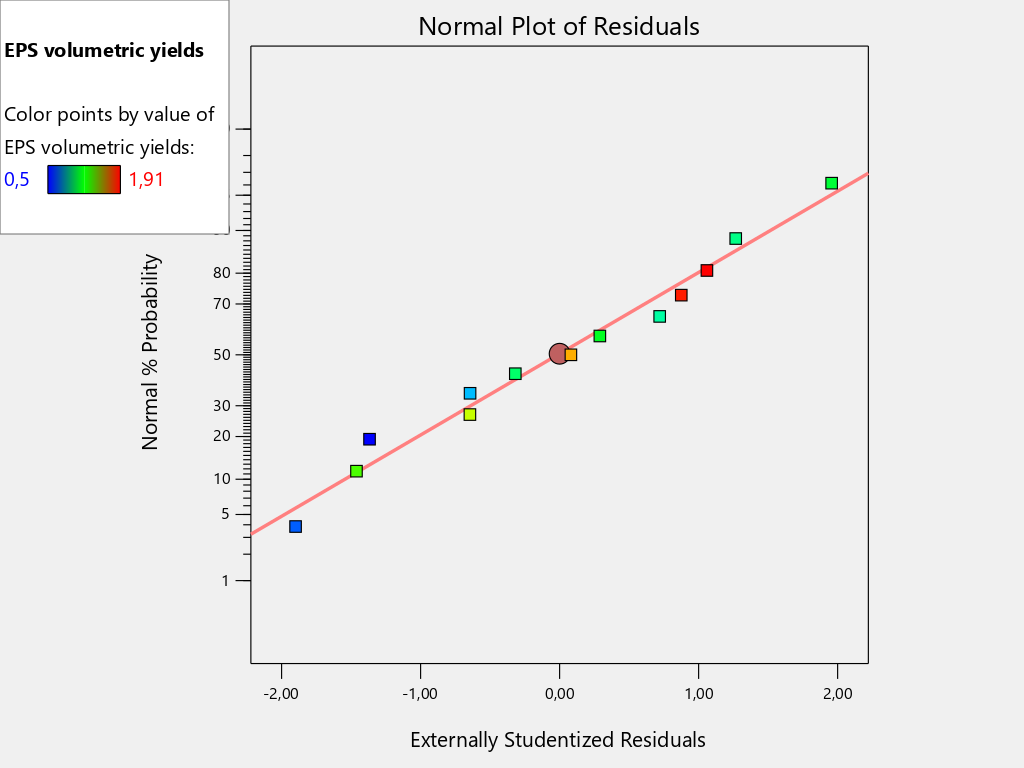


Figure S2: Normal plot analysis for the EPS production optimisation shows that the residuals were normally distributed.


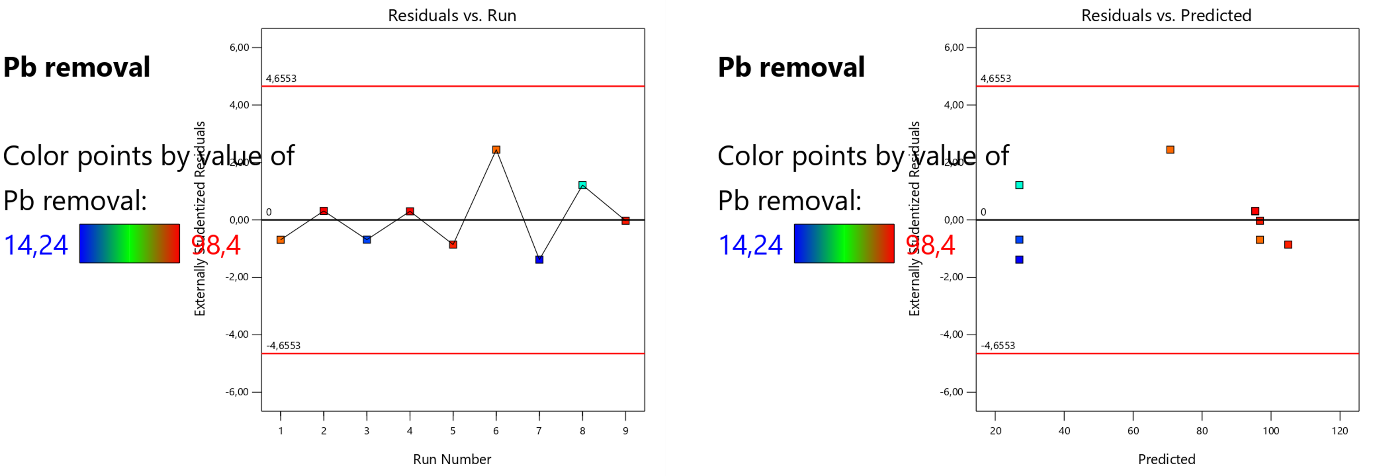


Figure S3: Model adequacy test for the EPS-hydrophobicity components cocktail. The Residuals vs. Run plots shows that the model data does not violate the independence assumptions on errors. The Residuals vs. Predicted plot shows that the residuals are not related to the predicted responses.


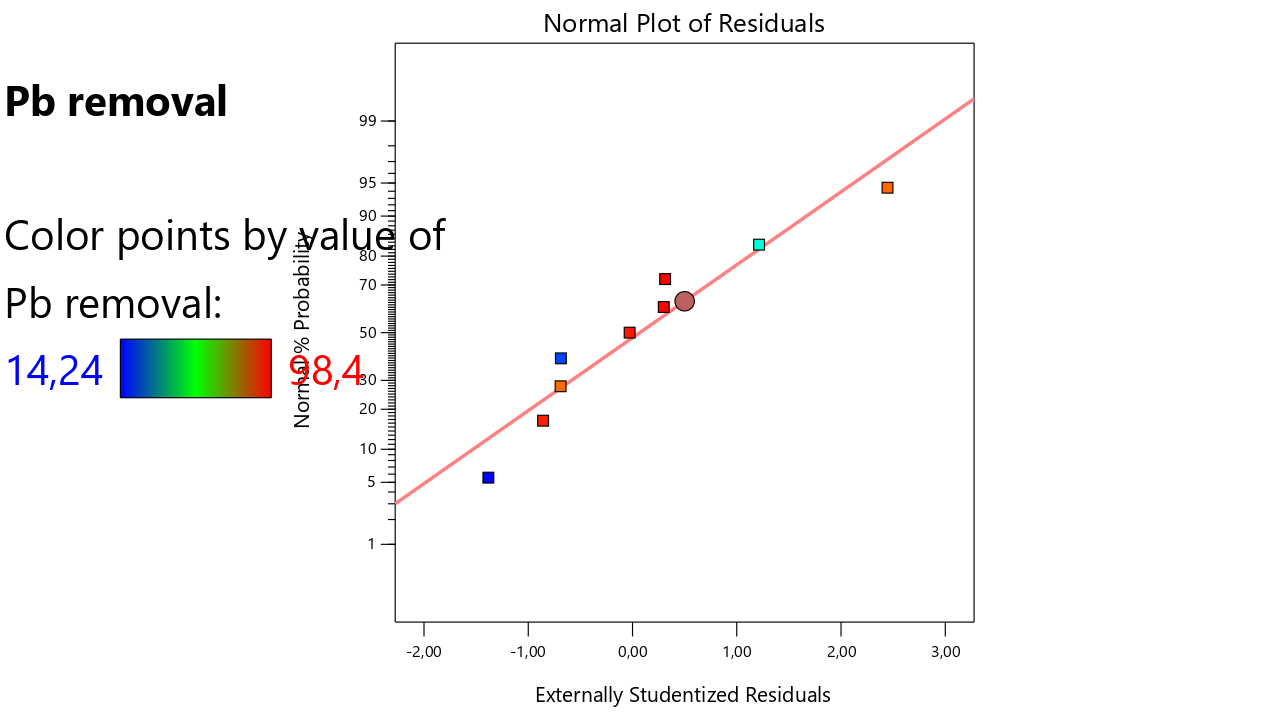


Figure S4: Normal plot analysis shows that the residuals for optimising the EPS-hydrophobicity components mixture were normally distributed.
